# Morphological Description, Phylogenetic and Molecular Analysis of *Dirofilaria immitis* Isolated from Dogs in the Northwest of Iran

**Published:** 2020

**Authors:** Majid KHANMOHAMMADI, Lame AKHLAGHI, Elham RAZMJOU, Reza FALAK, Reza ZOLFAGHARI EMAMEH, Kobra MOKHTARIAN, Mehdi ARSHADI, Minoo TASBIHI, Ahmad Reza MEAMAR

**Affiliations:** 1. Department of Parasitology and Mycology, School of Medicine, Iran University of Medical Sciences, Tehran, Iran; 2. Department of Laboratory Sciences, Marand Branch, Islamic Azad University, Marand, Iran; 3. Immunology Research Center, Iran University of Medical Sciences, Tehran, Iran; 4. Department of Immunology, School of Medicine, Iran University of Medical Sciences, Tehran, Iran; 5. Department of Energy and Environmental Biotechnology, National Institute of Genetic Engineering and Biotechnology (NIGEB), Tehran, Iran; 6. Medical Plant Research Center, Basic Health Sciences Institute, Shahrekord University of Medical Sciences, Shahrekord, Iran; 7. Al-Zahra Hospitals’, Tabriz University of Medical Sciences, Tabriz, Iran

**Keywords:** *Dirofilaria immitis*, Scanning electron microscopy (SEM), Homology, Iran

## Abstract

**Background:**

Dirofilariasis is a globally distributed arthropod-borne parasitic disease of mainly canids and felids. We evaluated to extend the knowledge of morpho-molecular characteristics and outer ultrastructure of *Dirofilaria immitis* isolated from Northwest of Iran.

**Methods:**

Overall, 67 filarial worms including 41 females and 26 males parasites were collected from the cardiovascular system of the 43 stray dogs in Meshkinshar, Ardebil Province, Northwest of Iran in 2017, and subjected to light and scanning electron microscopy (SEM) as well as carmine alum staining for morpho-molecular and identification. Molecular methods were used for confirmation of morphological findings by sequencing of Cyto-chrome c oxidase subunit I (*cox1*) gene.

**Results:**

The partial DNA sequencing of *cox1* gene of adult parasites showed considerable homology and close proximity to the previously isolated from Kerman and Meshkinshahr, Iran. The lowest genetic variation and the highest intra-species variability was found in *D. immitis* and *Dirofilaria repens*, respectively. No similarity was identified between *D. immitis* nucleotide sequence and *Wolbachia* species as its endosymbiont bacteria.

**Conclusion:**

The SEM technique is an excellent tool for differential recognition of the parasite surface morphology and molecular techniques could differentiate and identify *Dirofilaria* spp.

## Introduction

Dirofilariasis is a worldwide vector-borne filarial parasitism caused by *Dirofilaria immitis*. The worm is widely spread in the tropical, subtropical, and temperate regions and is considered as the main helminthic infection in the domestic and wild canines, felines and occasionally humans in endemic areas (1). This nematode usually localized in the cardiovascular system and causes serious disorders in definitive hosts (1, 2). The L1 larva develops in the mosquito vectors (from *Culicidae* family) into infective L3 larval stage. Inoculated microfilarias are circulated through the blood of the definitive hosts and migrating processes to the heart within six months (3). *D. repens*, *D. tenuis*, and *D. immitis* are the most commonly reported species in human subcutaneous infections (4). The first canine infection with *Dirofilaria* spp. in Iran was reported in 1969 (5). Several studies demonstrated filariasis of domestic and wild canids (6,7) and also a few reports of feline dirofilariasis in Iran (8, 9). In addition, 13 cases of human subcutaneous dirofilariasis have been reported from different parts of Iran as an emerging zoonotic infection (10).

The laboratory diagnosis of dirofilariasis in animals is usually based on direct microscopic observation of microfilariae. However, verification and reliable diagnosis of dirofilariasis are mainly dependent on standard serological and molecular analysis. Sometimes, the blood of infected dogs does not contain microfilariae, termed as amicrofilaraemic (occult) state infection (11). Molecular-based techniques provide an alternative approach with suitable sensitivity and specificity for the accurate identification of filarial parasites (12–15). Microscopic and serologic techniques are prone to false-negative. Many commercial kits, including ELISA and rapid dipstick methods, are available for serodiagnosis of dirofilariasis in dogs; however, the main problem is cross-reactivity with other parasitic infections, commonly found in domestic dogs (12, 16–18). The developed new rDgK antigen is sensitive (92.5%) and specific (87.5%) for immunodiagnosis of canine dirofilariasis using ELISA test (19). The differentiation between various *Dirofilaria* spp. by serological methods is almost impossible and usually requires more reliable methods. The development of an economical and robust method for identification of the known *Dirofilaria* spp. is morphological methods.

Herein, the purpose of this systematic evaluation was to extend the knowledge of morpho-molecular characteristics and outer ultrastructure of *D. immitis* isolated from Northwest of Iran.

## Materials and Methods

### Parasite sampling

This study was performed on 43 stray dogs (*Canis familiaris*) in Meshkinshar, Ardebil Province, Northwest of Iran (38° 23′56″N 47° 40′55″E) in 2017, which is a main endemic area for canine dirofilariasis in Iran. The serums of dogs were analyzed using an ELISA kit (DiroCHEK®, USA) and dipstick (SNAP®, CHW II, IDEXX, USA) according to the manufacturers’ instructions.

Six hyper-infected dogs, given decisive diagnosis, were necropsied and worms were collected from the cardiovascular system of the dogs with the permission of the Animal Ethics Committee of Iran University of Medical Sciences (IR.IUMS.REC1395.9221577203-2016.05.09).

### Direct microscopic study

We examined blood samples from 43 dogs with obvious symptoms of dirofilariasis and found that 27 dogs were *Dirofilaria* seropositive (62.8% CI: 47.9 to 75.6). Eight females and 16 males of *Dirofilaria* spp. were prepared and stained Carmine Alum (Sigma-Aldrich, USA) according to Gutierrez method (20). Hematoxylin and eosin (H&E) staining techniques were also applied on prepared sections from isolated parasites (21).

### Scanning electron microscopy (SEM)

Adult worms were fixed in 3% (w/v) ultra-pure glutaraldehyde (Sigma-Aldrich, USA), and immersed in 50 mM PBS pH 7.4 for 3 h at 4 °C, then five times rinsed with PBS during 30 min. Subsequently, worms were re-fixed in 1% (w/v) osmium tetroxide (Sigma-Aldrich, USA) in 50 mM PBS pH 7.4 for two hours, then washed and overnight remained in PBS. Parasites were gradually dehydrated using ethanol solutions, immersed in xylene and dried with critical point dryers (K850, Quorum, UK). Worms were stored under desiccation at 23±2 °C until further processing for SEM. Specimens were mounted onto stubs by conductive double-sided adhesive tape, sputter-coated with a thin layer of gold by Emitech SC7620 (Quorum, UK) and viewed by SEM (AIS2100, Seron, South Korea) (22, 23).

### Molecular-based analysis

Total genomic DNA of 24 *Dirofilaia* worms including eight females and 16 males were extracted from approximately 25 mg of each sample using a commercial DNA extraction kit (QIAGEN, Germany) according to the manufacturer’s instructions.

The species-specific mitochondrial *cox1* gene of *D. immitis* was amplified by primers as described (24) in 20 μl final volumes using 2X PCR Master Mix (RED Ampliqon, Denmark) and 1 μl of DNA template under following condition: 94 °C (5 min), [94 °C (30 sec), 52 °C (45 sec), 72 °C (60 sec)] × 30 cycles, 72 °C (7 min). DNA extracted from identified *D. immitis* by Prof. Mobedi and distilled water was applied as positive and negative controls, respectively. The PCR products were visualized on 1.5% agarose gel. The PCR purified product was sequenced in both direction and the representative submitted to GenBank under accession numbers MF288560. The phylogenetic analysis was performed by MEGA7 software using the maximum likelihood algorithm based on the Tamura-Nei model.

## Results

### Direct microscopic study

**Fig. 1:**
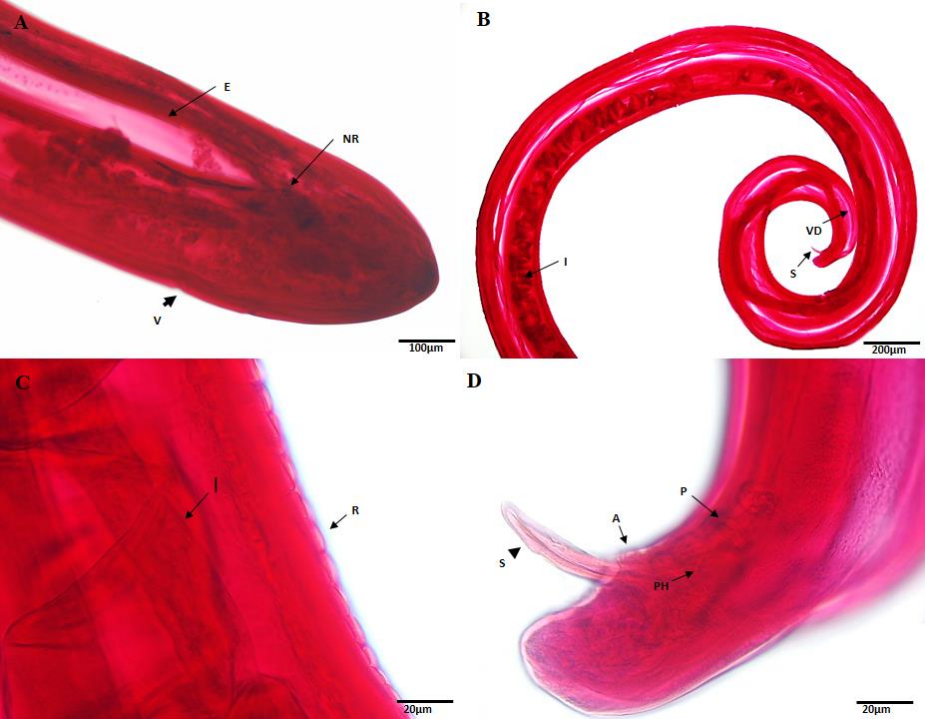
The characteristics of *D. immitis* by light microscopy (A–D, Alum carmine). (A) Cephalic end of male, apical view. Region of oesophagus and encircling nerve ring, respectively (thick arrow indicates vulvar pore); (B) Posterior part of male worm, lateral view, intestines, large spicule, and efferent channels showed by arrows; (C) Middle part lateral view of the worm, longitudinal segmented cuticular striations and intestine indicating by arrows; (D) Posterior part of male worm, lateral view, pre-cloacal papillae, anus, spicules and preanal supplementary organ are shown by arrows, respectively (arrow shows unequal round ended spicules). **Abbreviations:** A: anus; R: ridge; E: esophagus; I: intestine; S: spicule; NR: nerve ring; P: papillae; Ph: phasmid; V: vulva; VD: vas deferens

Twenty-seven out of 43 suspicious dogs were *Dirofilaria* seropositive (62.8%; 95% CI 47.9–75.6). Sixty-seven filarial nematodes, including 41 females and 26 males were obtained from necropsied dogs. The overall morphology of these adult worms was cylindrical with elongated gray whitish body transversely striated cuticle and males were smaller than females. The tails of the females were straight, large and rounded, while the tails of males were coiled. The cephalic portion was radially symmetrical and rounded (Fig. 1A). The parasites had specialized mouthparts; oral aperture did not contain any lips in the aring; mouthpart was surrounded by four pairs of small cephalic papillae with a pair of lateral amphids. Oesophagus consisted of two parts including short and spacious muscular (corpus) pharyngeal portion and a wide glandular area (Isthmus) along with marginal glandular cells. The posterior part of the oesophagus was moderately rounded and nerve ring encircled about 1/3 of the longitudinal section of the oesophagus. In Fig. 1A, the thick arrow shows a vulvar apparatus at the oesophagus– intestine junction. The anterior part of intestine was expanded. Although the indentation of the worm which was detectable below the head and near the esophagus, lateral deirids were not well-developed and situated at about the mid-level of oesophagus. No difference was observed between anterior ends in both sexes. The bottom of the male worms was spiral and entirely twisted.

The inequality of size and shape of the spicules was obvious (Fig. 1B). The salient features for morphological identification of the parasite were displayed in Fig. 1C by the cuticular longitudinal annulation along the parasite body and visible at the lateral view. The intestine of the parasite was visible as a zigzag shape, based on the consumed food. The specialized structure of the tail of adult worm was defined and included two pairs of pre-cloacal papillae as well as three large pairs of ventro-lateral post-cloacal papillae. The pair of small lateral phasmids located slightly toward the anterior of the first pair of lateral preanal papillae (Fig. 1D). In this portion, two well-sclerotized asymmetrically round ended spicules with approximately 110–128 μm size were present. In the posterior part of worms, four pairs of bilateral featured papillae were found. The anus was situated in the back of spicules of ventral posterior.

**Fig. 2:**
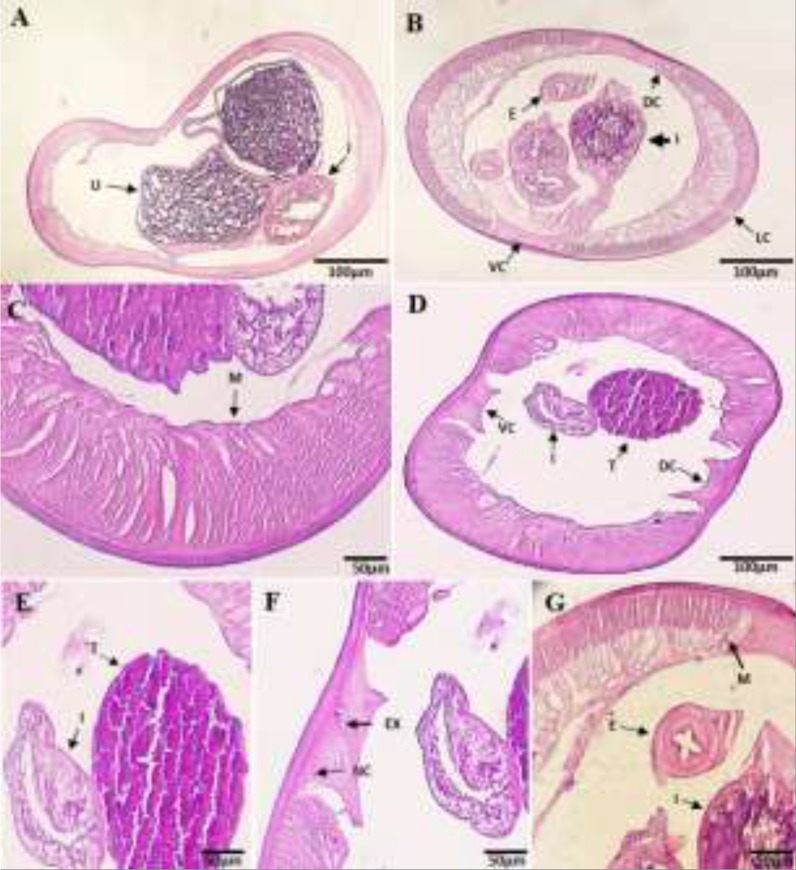
The characteristics of adult *D. immitis* cross section (A–G, H&E). (A) Sectional view of female worm; arrows indicate distended uterus filled with eggs and intestine; (B) Sectional view of male worms, muscular part of esophagus, intestine and two pairs of dorso-ventral cords (see arrows); (C) Arrow indicates cross section of longitudinal coelomyarian tall muscle fusiform cells; (D&E) Granular testis, portion of intestine and trapezoidal cords showed by arrows; (F) Expanded multi-layered cuticle, large lateral chords, nerve and execratory canal (see arrows); (G) Muscular part of esophagus intestine with food lump represents with arrows. **Abbreviation:** CL: Cuticular Layer; DC: dorsal cord; E: esophagus; I: intestine; LM: Longitudinal muscle; LC: lateral cord; ML: muscular layer; NC: nerve canal; T: testis; VC: ventral cord; U: uterus

The transverse sections investigated by light microscopy. These nematodes showed a tubular morphology and circular cross-section. Paying attention to Fig. 2A revealed that pseudocoelom distended uterus is filled with many thin-walled embryonated eggs and intestines with food lump (see arrows). Fig. 2B demonstrates muscular part of the esophagus, dorsal and ventral hypodermal cords, and granular testis. In Fig. 2C, multi-layered cuticle, basement membrane irregular thickening, and tall coelomyarian muscle fusiform cells under the basal membrane were shown. The cross-section of intestine and pair of trapezoidal hypodermal cords in pseudocoelom are shown in Fig. 2D. In Fig. 2E, a cross-section of the male worms is shown, which demonstrates the dense and granular testis along with a section through the intestine. In Fig 2F, the ventral hypodermal cord, central excretory canal, and lateral nerve canal were demonstrated with arrows. In Fig. 2G, the arrangement of the strong circular muscle of oesophagus and the characteristic features of the arrangement of lateral longitudinal muscles, expanding into both narrow hypodermal chords observed. The muscular arms extends to both ventral and dorsal nerve cord, designed for the sinusoid-like motions.

### Scanning electron microscopy (SEM)

**Fig. 3:**
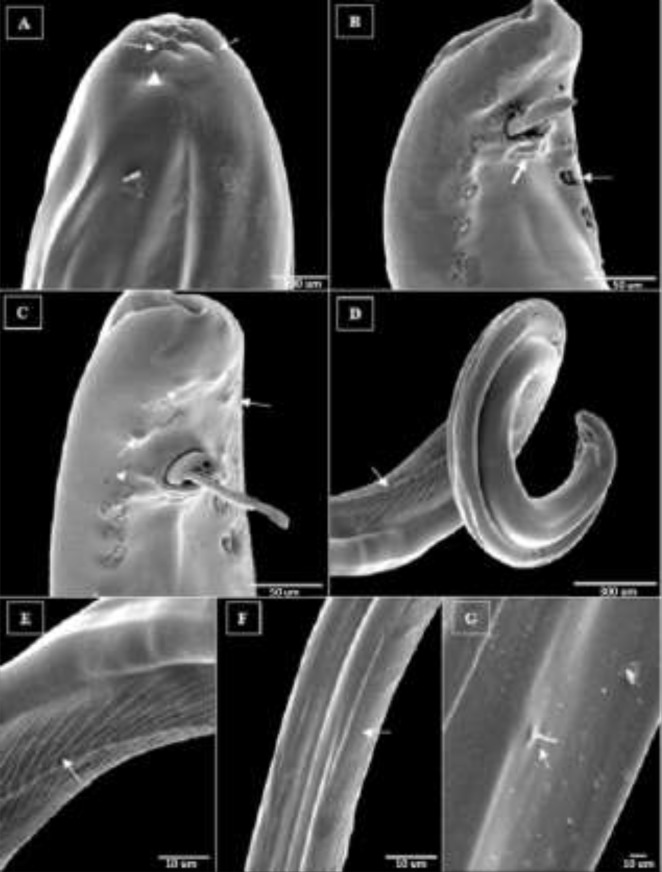
Scanning electron micrographs of male *D. immitis* (A–G). (A) The cephalic end of male, apical view, details of mouth, supplementary organization and two thin arrow indicates a pairs hole, lateral amphideal fovea showed by arrow head; (B) Posterior portion, ventral view, 4 pairs longitudinal slot (valve shape) papillae’s, anus and two unequal spicules (see arrows); (C) Posterior portion, ventral view, five pairs of post-cloacal bump shown by arrows, heat of arrow indicate phasmids, thin and longs spicule is seen; (D) Posterior part of body, ventral view, arrow indicate longitudinal striations and interstitial of annuli; (E) Middle part of body (rugosa area), ventral view, the interstitial distance of annuli gradually will be longer; (F) Posterior part, the caudo-dorsal view, dorsal wrinkled, and fine longitudinal cuticular discontinuous striations shown by arrow; (G) Dorsal part of body, the periphery bristle specifies by arrow

The SEM evaluation of the worms showed that the oral aperture was markedly rounded, surrounded by four outer and inner lip-like structures and the pairs of lateral amphids (Fig. 3A). Amphideal fovea situated within a distance from the cephalic apex, with a slot shape. A pair of sensory organs (amphids) located in the lateral interior region. The development of the cephalic vesicle was observed throughout cervical papillae region. Two small holes are remained in the cephalic ciliary channel and central canal on the anterior end. Four pairs of valve-like pre-cloacal papillae and the anus orifice were shown with the thick arrow below the spicules (Fig. 3B). At the caudal end of the male worms, two asymmetrical well-sclerotized spicules were present. The large spicule is strong, and nearly straight with round tips and has covered the shorter spicule.

Figure 3C shows five pairs of mammary bumps shaped under the spicules. Region of cloacal opening elevated and one pair of large subventral preanal papillae and two pairs of small postanal papillae situated on two large subventral postcloacal sides. Two nearly parallel papillae were located close to each other, posterior to cloacal orifice. Straightly on both lateral sides of spicules, a pair of embedded button shape amphids were visible. This position demonstrates small spicules with leaf shape and sharp tips (Fig. 3C). The body is often ornamented with ridges and fine transverse striation in the cuticle and can be seen at the posterior end. The pattern of ridges and striations starting from the middle part on the body to the spiral tail region got longer at the distant regions; Longitudinal ridges are more developed on the ventral surface than on the dorsolateral position (Fig. 3D). The surface of cuticle in SEM was constructed by transverse annular longitudinal ridges of the rugosa area with an interstitial distance of approximately 0.4–0.11 μm throughout the body. In the ventral view of the body, the interstitial distance of annuli in the posterior end of body gradually got longer (Fig. 3E). In the dorsal position and both sides of the worm, fine discontinuous longitudinal striations are found (Fig. 3F). Caudal periphery was thickened caused by technical errors during the dehydration process or other preparation procedures of the specimens and there was no other accessory structure in this area. In the dorsolateral part of body, a periphery bristles with an outstanding oval base was present (Fig. 3G).

### Molecular-based analysis

**Fig. 4:**
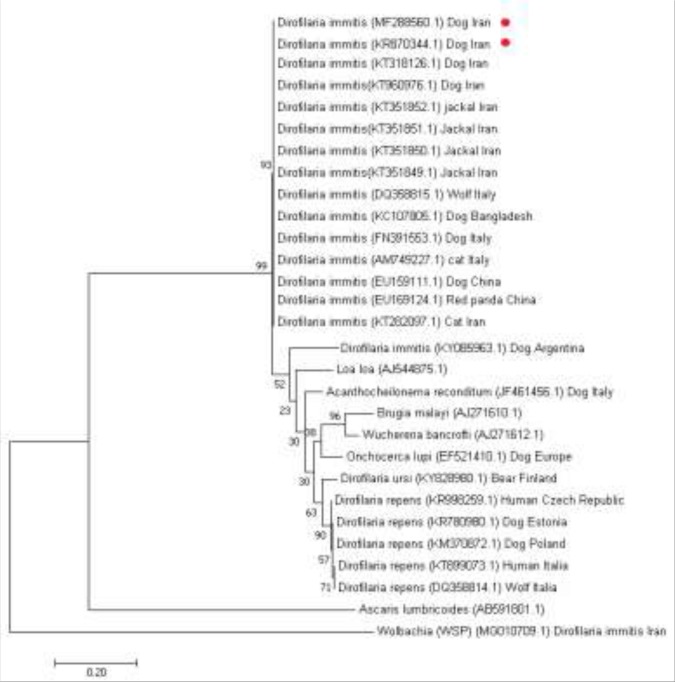
Phylogenetic tree of *D. immitis* cytochrome c oxidase subunit I (*cox1*) gene. The tree was drawn using maximum likelihood (ML) analysis of the sequences based on the Tamura-Nei model with 1000 bootstrap repetition. The scale bar indicates the genetic distance in single nucleotide substitutions. *Ascaris lumbricoides* was used as out group

The phylogenetic analysis was carried out using *cox1* gene sequences, obtained during this study (MF288560) and compared with sequences available in GenBank (https://www.ncbi.nlm.nih.gov/genbank). The Basic Local Alignment Search Tool (BLAST) homology analysis indicated maximum homology with *D. immitis* isolated from different hosts (Fig. 4). The molecular characterization of all involving *cox1* genes from *D. immitis* isolates showed 100% homology with sequences already annotated in NCBI database from other geographical areas in the world.

## Discussion

In the present study on *D. immitis*, we tried to confirm the correlation of histomorphological characteristics using the molecular and SEM studies. There are limited SEM studies available on *Dirofilaria* genus (23, 25, 26). Furthermore, the limited reports from Iran were mostly based on the detection of this worm by light microscopy (6). Interestingly and within basic diagnosis of *Dirofilaria* genus, the cephalic part was very small and rounded but had active functionality. Two pairs of small holes were found in this region and seemed to be the remnants of the cephalic ciliary channel and the central canal. These canals play a functional role in the development of immature forms of adult ones (27). The morphological and cuticular characteristics of five *Dirofilaria* spp.; *D. immitis*, *D. repens*, *D. tenuis*, *D. corynodes,* and *D. magnilarvatum* first described by Wong et al (28). Other similar patterns including the body dimensions and shape of the oesophagus of both sexes, cephalic portion, the morphology of spicules, circumstances of amphids, the position of the vulva pore to the oesophago-intestinal junction, the surface structure of the cuticle, transverse longitudinal and annular striations pattern, location of the deirids, lateral line, and the morphology, as well as the number of caudal papillae’s, were observed. Our SEM observations of the transverse longitudinal cuticular annular striations along with the entire length of body confirm the previous findings. SEM and the molecular characterization of *D. immitis* isolated from leopards and penguins in a Japanese zoo confirmed that the parasites belonged to *D. immitis*, commonly found in canids (23, 29).

In *D. immitis*, the ridges and striations pattern of the ventral portion of the spiral tail in the male was dramatically artistic. This is a special pattern for *D. immitis* and was unlike those of other *Dirofilaria* spp. (26). In the current study, the morphological characteristics were compatible with the descriptions of the posterior and anterior regions of the male worms such as distribution of caudal papillae and basically confirmed the reports from previous researchers (25, 26, 30, 31). In contrast, morphological comparison among male worms isolated from dogs showed that the ventral ornamentation of the posterior zone (rugosa) was similar between all studied samples. Notably, the location of male phasmids and number of precloacal papillae’s of the studied worms showed a significant difference with reports of Sano et al. (29). Therefore, distribution of precloacal and post-cloacal papillae is not a reliable morphological taxonomic specification for diffraction of *D. immitis* (25). We observed four and five pairs of pre- and post-anal papillae in male parasites, respectively (23, 25, 26, 31). Other morphological characteristics were compatible with previously reported definitions of the parasite, including the apparent size and accessory structures (6, 9, 25, 26, 30). SEM studies showed no significant difference between anterior ends of both sexes in the papillae’s and cuticular pattern.

The phylogenetic analysis of *cox1* gene confirmed *Dirofilaria* species in Kerman and Meshkinshahr was identical, also revealed the highest homology of *D. immitis* isolated from dogs (KR 870344 KT960976, KT318126) and jackal (*Canis aureus*)(KT351850, KT351851, KT351852) in Iran, as well as China (EU159111), Italy (FN391553), and Bangladesh (KC107805). A significant homology showed with cat in Iran (KT282097) and Italy (AM749227), wolf from Italy (DQ358815), and red panda from China (EU169124).

The pairwise homology analysis revealed that the isolated worms showed maximum similarity with the available DNA sequences at NCBI GenBank. These findings demonstrated the low genetic variability among isolated *D. immitis*; on the contrary, *D. repens* shows more intra-species variability, which subject correlation with its biological function. Only a minimal genetic variation may have occurred among *D. immitis* isolates. The comparison of *cox1* gene from Meshkinshahr isolated with those retrieved from GenBank showed remarkable nucleotide dissimilarities with the related gene sequences of genus isolated from dogs in Argentina (KY085963) as well as other filarial nematodes (32). There was lower intra-species variability in *D. immitis* isolateds in this study. The most intra-species variability belonged to *D. repens,* which made it as a distinct species (6, 9, 33, 34). *Wolbachia* spp. can be the endosymbiont bacterium and are found in filarial nematodes (35). In the current molecular study, the analysis did not show any similarity between *D. immitis* nucleotide sequence with this alpha protobacterium. The main benefit of a PCR-based technique is in specific identification and differentiation within different species, especially in the existence of the mixed infection (33–36). Thus, the development of an appropriate diagnostic method for description and differentiation of *Dirofilaria* spp. could be helpful.

## Conclusion

Due to having specific characteristics in this parasite, morphological methods are valuable and could increase our understanding of early diagnosis of dirofilariasis. SEM technique is an excellent tool in recognition of the parasite surface morphology. Since PCR has extensive applications in the molecular-based diagnosis of parasitic infections and could help us in differentiation and identification of all *Dirofilaria* spp. as well. Nevertheless, further complementary studies are recommended for detailed identification of *Dirofilaria* spp.
